# The Effects of Biochar on Indigenous Arbuscular Mycorrhizae Fungi from Agroenvironments

**DOI:** 10.3390/plants10050950

**Published:** 2021-05-10

**Authors:** María Videgain-Marco, Pedro Marco-Montori, Clara Martí-Dalmau, María del Carmen Jaizme-Vega, Joan Josep Manyà-Cervelló, Francisco Javier García-Ramos

**Affiliations:** 1 Departamento de Ciencias Agrarias y del Medio Natural, EPS, Universidad de Zaragoza, Carretera de Cuarte s/n, E-22071 Huesca, Spain; cmarti@unizar.es (C.M.-D.); fjavier@unizar.es (F.J.G.-R.); 2 Centro de Investigación y Tecnología Agroalimentaria de Aragón (CITA), Instituto Agroalimentario de Aragón—IA2 (CITA-Universidad de Zaragoza), Unidad de Recursos Forestales, Avenida Montañana 930, E-50059 Zaragoza, Spain; pmarcomo@cita-aragon.es; 3 Departamento de Protección Vegetal, Instituto Canario de Investigaciones Agrarias (ICIA), Carretera de El Boquerón s/n, Valle Guerra, La Laguna, E-38270 Tenerife, Spain; mcjaizme@icia.es; 4 Thermochemical Processes Group, Aragón Institute of Engineering Research (I3A), EPS, University of Zaragoza, Carretera de Cuarte s/n, E-22071 Huesca, Spain; joanjoma@unizar.es; 5 Instituto Agroalimentario de Aragón—IA2 (CITA-Universidad de Zaragoza), EPS, Universidad de Zaragoza, Carretera de Cuarte s/n, E-22071 Huesca, Spain

**Keywords:** vine-shoots, sorghum, lettuce, drought stress, trap plant, waste management

## Abstract

The effects of biochar on soil–plant–microorganisms systems are currently being extensively investigated. Considering that arbuscular mycorrhizal fungi (AMF) play an essential role in nutrient dynamics, the present study aims at understanding vine shoot-derived biochar effects on AMF activity and the impact of their multiplication in soils on water-stress resistance of plants. Three agronomic tests were performed in greenhouse pots. The first experiment evaluated the effects of three factors: final pyrolysis temperature for biochar production (400 °C and 600 °C), application rate (0 weight-wt.- % as a control, 1.5 wt. %, and 3.0 wt. %) and texture of the growing media (sandy-loam and clay-loam origin) on AMF, microbial communities and phosphatase activity. In the second experiment, an indigenous consortium of AMF was multiplied through the solid substrate method and sorghum as a trap plant with biochar addition. This process was compared to a control treatment without biochar. Obtained inocula were tested in a third experiment with lettuce plants under different water irrigation conditions. Results from the first experiment showed a general increase in AMF activity with the addition of the biochar produced at 400 °C in the sandy-loam texture substrate. Results of the second experiment showed that the biochar addition increased AMF root colonization, the number of AMF spores and AMF infective potential. Results of the third experiment showed that biochar-derived AMF inoculum increased AMF root colonization, AMF spores, dry biomass and the SPAD index in a lettuce crop under low-water irrigation conditions.

## 1. Introduction

New crop varieties and biotechnologies are being investigated in dealing with drought-stress events in agriculture [1]. Concurrently, indigenous crop varieties and their adaptation mechanisms have been valued as agroecological strategies that contribute to improving drought resistance in rainfed areas. Improving the physical and biological fertility of the soil is becoming a mandatory practice to preserve its productive capacity, even though chemical fertilization remains the focus of intensive agricultural systems.

Currently, one of the most studied organic amendments to enrich soil fertility is biochar, a carbon-rich product obtained by thermal degradation of biomass under a limited supply of oxygen, at temperatures usually below 700 °C, which is produced to be added to the soil as a means of improving its quality and increasing carbon storage [2].

Previous studies have already reported that biochar is highly recalcitrant and able to improve some soil properties by influencing biochemical and biological processes [2,3,4,5]. Biochar amendment significantly enhances the nutrient availability and nutrient retention of a wide range of soils [6], in addition to the positive contribution to the improvement of other physical and biological soil properties [7,8,9,10] and to metal retention [11]. However, the capacity of this material to provide or enhance soil fertility extremely depends on the type of feedstock and pyrolysis conditions [12].

The effects of biochar (when it is used as a soil amendment) on the soil–plant–microorganisms system are currently being extensively investigated. Since microorganisms play an essential role in soil nutrient dynamics, they can be used as bioindicators due to their high sensitivity to small short-term habitat modifications. Furthermore, microorganisms have high resilience to degradation processes and biotic/abiotic stresses [13].

Droughts are a limiting factor in agricultural production. In addition, due to climate change, droughts are increasing in frequency and severity in some regions [14]. Alleviation of this abiotic stress through the enhancement of the arbuscular mycorrhizal fungi (AMF) presence in soils could be an interesting agroecological strategy, since AMF can shape the adaptative strategy of plants. The most important mechanisms by which symbiosis can alleviate drought stress in host plants are related to the direct uptake of water through the fungal hyphae, changes in soil water retention properties, better osmotic adjustment of AM plants, enhancement of plant gas exchange and water-use efficiency, and protection against oxidative damage generated by drought [15,16].

Research studies involving biochar and AMF processes show a wide spectrum of results, depending on experimental conditions, biochar composition and particle size [17]. The indigenous AMF consortia in soils are usually selected as bioindicators, since they are considered crucial components of soil fertility, either due to symbiotic relationships with plant roots or interactions with rhizosphere microorganisms [18]. Some earlier studies reported negative effects of biochar addition on AMF root colonization for both indigenous AMF consortia and introduced species. Warnock et al. [19] reported a decrease in the AMF abundance with wood-based biochar addition accompanied by changes in phosphorous availability. In line with this, several research studies reported negative or no significant effects [20,21] of biochar on AMF development. But there is a clear interaction between biochar, microorganisms, mineral nutrients available in the soil and water conditions. LeCroy et al. [22] showed that biochar reduced biomass production of sorghum plants in the presence of high levels of nitrogen fertilizer; they suggested that soil nitrogen controls the ability of char to influence the mycorrhizal symbiosis and the degree to which the fungi oxidize the char surface. Vanek and Lehmann [23] found that positive biochar-AMF interactive effects on bean phosphorus (P) uptake were fostered by sparingly soluble Fe-P; however, they were not observed when soluble P was combined with biochar. In this sense, Blackwell et al. [24] and Solaiman et al. [25] also correlated AMF increases to low nutrients availability in soil. Hammer et al. [26] observed AM fungal hyphae accessing microsites within biochar mediating plant P uptake from the biochar surface.

Considering the wide range of results reported in the literature, the possibility of using biochar as a component of the growing media in containerized plant production [27] and its utility as a potential carrier material for delivery inoculants [28], assessing the suitability of biochar as a component of the growing media on the multiplication process of an indigenous consortium of AMF is proposed herein. For this purpose, a first bioassay was conducted in pots under controlled greenhouse conditions. Sorghum (*Sorghum bicolor* L. Moench) was selected as a classic mycotrophic test crop to evaluate the effect of the addition of two types of vine shoot-derived biochar on the AMF activity and other biological indicators (i.e., phosphatase activity and microbial community variations). The experiment measured the influence of different factors (the final pyrolysis temperature at which the biochar was produced, application rate and growing media substrate texture). Based on the results of the abovementioned bioassay, it was hypothesized that biochar could be suitable as a component of the growing media on the multiplication process of indigenous AMF consortia in soils through the method of solid substrate and trap plants [29]. This approach was tested experimentally, and the final inoculum obtained was then evaluated in terms of water-stress resistance for lettuce (*Lactuca sativa)* plants, which were grown in the same soil under study.

## 2. Results

### 2.1. Biochar Properties

The complete characterization of the two types of biochar produced is detailed in a previous publication [30]. The results were primarily dependent on pyrolysis operating conditions. The biochar average mass yield (ƴ_char_) notably decreased when the pyrolysis final temperature increased (biochar at 400 °C—ƴ_char_ = 0.38; biochar at 600 °C—ƴ_char_ = 0.29). The biochar produced at 600 °C (B600) had a higher fixed carbon content measured on a dry basis (84.54 wt. %) and ash (10.02 wt. %) compared to biochar produced at 400 °C (B400: 70.88 wt. % and 6.45 wt. % respectively), which conversely had a higher amount of volatile matter (+19.64 wt. %). The specific surface area and pore volume increased with rising pyrolysis final temperature. Related to it, water-holding capacity (WHC) increased with pyrolysis final temperature, from 14.16 v.% in B400 to 18.35 v.% in B600. Principal and secondary nutrient levels showed no major differences between B400 and B600. Table S1 (Supplementary Material) details the results from proximate, elemental and physicochemical analyses of the biochar produced.

### 2.2. Soils Characterization

Results from the AMF infective potential of soils and microbiological analyses are summarized in Table 1.

Student *t*-test did not show statistical differences between Soil 1 and Soil 2 for the biological characteristics analyzed.

The AMF infective potential showed mean values of 39.6 and 30.9 propagules in 100 cm^3^ of soil, for sandy-loam soil (S1) and clay-loam soil (S2), respectively. The initial mean number of AMF spores was 420 spores in S1 and 465 spores in S2. The morphological identification of the spores reveled that *Glomus* spp. was the main species identified in the samples of both soils (77% in S1 and 94% in S2). This species is largely found in natural and cultivated ecosystems and stands out for its high efficiency on a large number of crops and its adaptation to a wide pH range [31]. In addition, it was detected, in lower percentages, the availability of other species such as *Gigaspora* spp. (20% in S1), *Scutelloespora* spp. (3% in S1) and *Acaullospora* spp. (6% in S2).

With respect to the culturable microbial communities, soils achieved an average initial total microbial count, mesophilic aerobic microorganisms (MAM), of 7.5 and 8.0 log cfu g^−1^, for sandy-loam and clay-loam substrates, respectively. The predominant microbial group in both soils was actinomycetes (ACT) (6.1 and 6.5 log cfu g^−1^, respectively for each substrate), followed by *Pseudomonas* genus (PS) (5.7 and 6.0 log cfu g^−1^, for sandy-loam and clay-loam substrates, respectively). The mycobiota (molds and yeasts) was present at relatively lower numbers with average counts of 4.7 cfu g^−1^ for both substrates.

Results from physicochemical fertility of Soil 1 and Soil 2 are shown in Table S2 (Supplementary Material). The main differences between both samples were found in the content of oxidable organic matter (S1: 3.04 wt. %; S2: 1.70 wt. %), macroelements content (mg kg^−1^: N-NO_3_: S1—59, S2—12; P Olsen: S1—32, S2—29; K: S1—160, S2—252) and WHC (S1—5.41 v. %; S2—10.22 v. %).

### 2.3. Experiment 1: Effects of Soil Texture, Addition Rates and Final Pyrolysis Temperatures on AMF Performance, Microbial Communities’ Variation and Phosphatase Activity

During crop development, a general increase in root AMF colonization was observed from 210 to 330 days after sowing (D_210_ and D_330_); the average values of AMF root colonization decreased in the last period, which could be related to winter temperatures and the final development of the crop. The average values measured and standard deviation for this parameter and the number of AMF spores at the final of the experiment are summarized in Table 2.

No differences on root colonization were observed for the measurements performed during the first seven months of the experiment (from D_120_ to D_210_) in either of the two types of growing media.

Significant differences in root colonization were observed in measurements made from D_330_. For both sandy-loam and clay-loam substrates, the highest percentage of colonization was observed in the treatment with B400 addition (20.0% and 31.7% for S1 and S2 respectively), although there were no differences in comparison to Control treatment for the clay-loam substrate. This trend between the treatments was maintained up to the last measurement date (D_390_) with lower average colonization values, as mentioned above. It should be noted that there was a marked decline of AMF root colonization in B600 treatments with lower doses in S1 (from 10% to 5%) and with high doses in S2 (from 18.3% to 6.7%) at the end of the experiment (D_390_).

The number of AMF spores at the end of the experiment (D_390_) had significantly different results between treatments (see Table 2). In particular, a clear influence of biochar addition in the sandy-loam substrate in comparison to Control treatment was observed. The highest value in this substrate was reported in B400 at high doses of biochar addition (1511 spores per 100 g of soil) with no significative differences with respect to B600 treatments (1380 and 1095 spores per 100 g of soil in D1 and D2 treatments, respectively). Concerning the clay-loam substrate, no differences were observed in the number of AMF spores between treatments with the exception of B600 at a high dose of biochar addition (5245 spores per 100 g of soil). Identified genera information is available in Table S3. No differences were observed between treatments for genera distribution at the end of the experiment.

**Figure 1:**
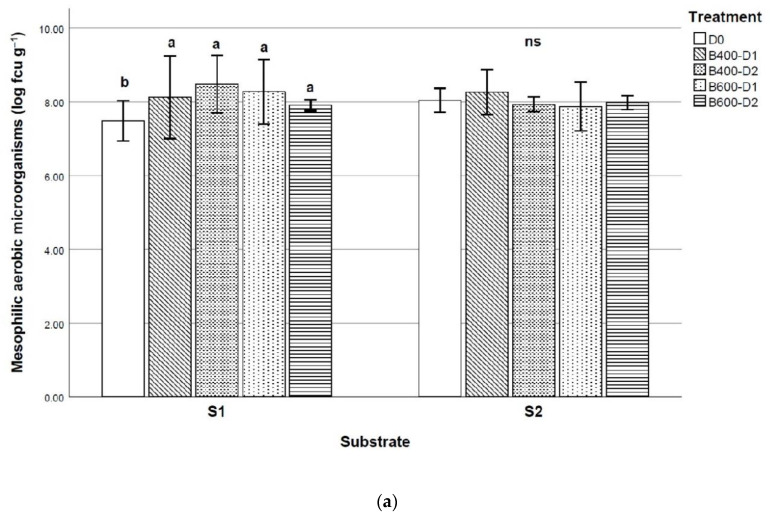

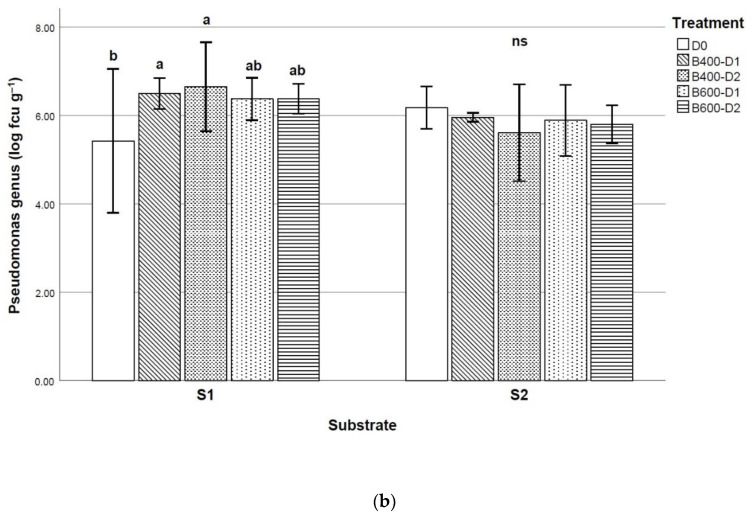
Effect of biochar application on (**a**) mesophilic aerobic microorganisms and (**b**) Pseudomonas genus. (Final pyrolysis temperature of biochar B400—400 °C and B600—600 °C; D0–Control without biochar, D1—1.5 wt. %, D2—3 wt. %; S1—sandy-loam substrate, S2—clay-loam substrate). Different letters show statistically significant differences at *p* ≤ 0.05 (Tukey’s test).

MAM and PS were the microbial communities in which significant differences were observed after biochar addition. As shown in Figure 1, the overall average value of MAM increased slightly from the initial counts, and the biochar application significantly increased the average values in the sandy-loam substrate, from 7.5 cfu g^−1^ in Control treatment to 8.2 cfu g^−1^ after biochar addition. For this texture soil, PS counts showed the highest values when B400 was added. In S1, B400 and B600 showed similar effects. B400 increased the average value in comparison to Control treatment for this texture soil. However, no differences were observed for these microbial communities under biochar addition in the clay-loam substrates.

Complete counts of the mesophilic aerobic microorganisms, *Pseudomonas* genus, actinomycetes, and mycobiota (yeasts and molds) are detailed in Table S3. The statistical analysis of microbiological counts performed in substrates at the end of the experiment (D_390_) did not show significant differences in actinomycetes and mycobiota populations. The final values were similar to those measured during the initial characterization.

For the specific results of our experiment, no differences in P content were found on substrates or plant tissue [30]; however, a positive correlation between AMF activity and *Pseudomonas* abundance was confirmed for the sandy-loam substrate (see Table S4).

Enzymatic activities have an important function in organic P mineralization and in P plant nutrition, especially in calcareous soils subjected to frequent retrogression processes of available P [32]. For all the substrates and biochar application rates assessed herein, the AcdP activity values (1.38 µmol h^−1^ in average) were lower than AlkP ones (2.98 µmol h^−1^ g^−^^1^ in average). This finding, however, was somewhat expected, since microbial population in soils can be sensitive to the growing pH media of both soils sampled for substrate preparation.

**Figure 2:**
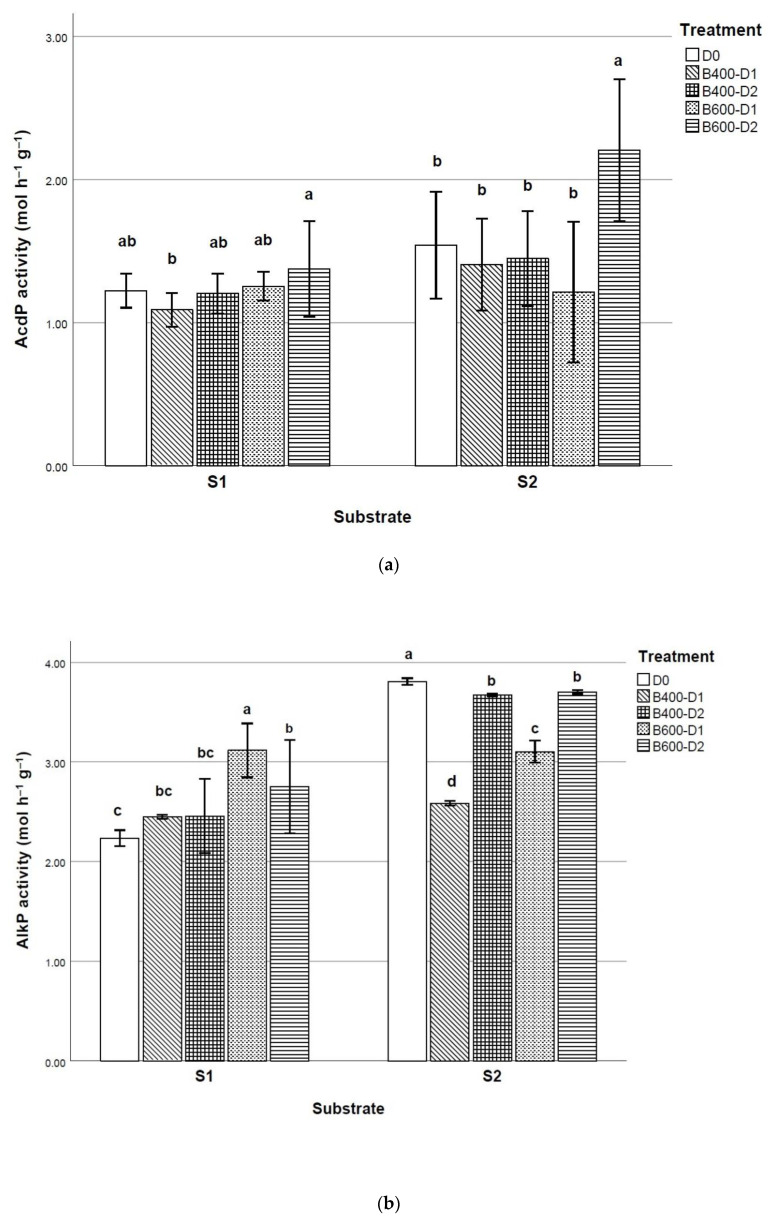
Effect of biochar application on (**a**) acid phosphatase activity and (**b**) alkaline phosphatase activity. (Final pyrolysis temperature of biochar B400—400 °C and B600—600 °C; D0–Control without biochar, D1—1.5 wt. %, D2—3 wt. %; S1—sandy-loam substrate, S2—clay-loam substrate). Different letters show statistically significant differences at *p* ≤ 0.05 (Tukey’s test).

From the analysis of the phosphatase activity in relation to substrate granulometry, it was deduced that, for the sandy-loam substrate, both AcdP and AlkP values had similar behavior (see Figure 2), leading to significant activity increases with the addition of B600 in comparison with B400. Both AcdP and AlkP values did not show significant differences between B400 and Control treatment in the sandy-loam substrate (S1).

Nevertheless, for the clay-loam substrate (S2), AcdP only showed significant differences in B600 treatment; in contrast, the highest main value of AlkP in S2 was obtained for the Control treatment without biochar, which had results significantly differents from treatments with biochar. In this texture (S2), the lower dose of biochar addition was related to a decrease in AlkP activity, both with B400 and B600 addition. The complete results are detailed in Table S3.

Table 3 shows the results from the three-way ANOVA on the significant effects of the factors (i.e., growing substrate texture, biochar final temperature, and application rate) on root AMF colonization at different dates after sowing (D_210_, D_330_ and D_390_), number of AMF spores, phosphatase activity and microbial communities at the end of the second crop development cycle (D_390_).

As can be seen from the results reported above, the growing-substrate medium was the most influential factor for the response variables assessed. Results obtained in S1 demonstrate that biochar addition had the greatest positive influence in fine texture, in terms of AMF root colonization, number of AMF spores and enzymatic activity. Biochar temperature also had an important effect, with a positive trend in AMF root colonization under B400 addition. Application rate had the lower effect considering it separately (only significant in enzymatic activities); however, significant effects were observed by the interactions with biochar on the studied variables.

Considering these results, B400 was selected as a component of the solid substrate used in Experiment 2 to multiply the AMF consortium present in the sandy-loam substrate (S1). The amount of biochar selected to prepare the multiplication substrate was 1.5 wt. %, since this concentration value could be relatively easily implemented in real field conditions.

### 2.4. Experiment 2: Multiplication Process of AMF Consortium on Solid Substrate with Biochar

The results of AMF root colonization (%), number of AMF spores and infective AMF potential of substrates at the end of the experiment are summarized in Table 4.

The results of this experiment followed the same trend as in Experiment 1, with significant increases (*p* ≤ 0.05) in all measurements carried out in T1 with respect to T0. Biochar addition increased AMF root colonization by 211%, AMF spores by 168% and infective AMF potential by 223% with respect to the treatment without biochar.

It should be highlighted that there were a relatively large number of AMF spores, regardless of the procedure adopted for their quantification (i.e., counts of their isolation through wet sieving and the most probable number of mycorrhizal propagules).

### 2.5. Experiment 3: Effects of Inoculum Application on Water-Stress Resistance and Development of Lettuce Plants

As expected, results obtained in Experiment 3 showed a strong interaction between water-irrigation conditions and AMF application (*p* ≤ 0.05) for all the parameters studied, with the exception of the SPAD index in D_60_. This interaction allowed us to analyze the data among the different scenarios of water contributions.

The results of AMF root colonization (%), number of AMF spores, dry biomass and the SPAD index at the end of the experiment are shown in Table 5.

During the first month of crop development, in which all the plants received the same amount of water, no differences were observed in the SPAD index among the treatments.

At the beginning of the second month (D_50_), in which the irrigation dose was reduced (i.e., half of the plants), the SPAD index showed differences between treatments only in those plants that received less water (WID2). Plants growing with AMF inoculum produced with biochar B400 (+B+AMF) showed a significantly higher SPAD index with respect to the plants inoculated with the inoculum produced in the conventional way (+AMF) under low-irrigation conditions. Furthermore, under this irrigation scenario, non-mycorrhized plants showed significantly lower values than mycorrhized plants.

During the period elapsed between D_50_ and D_62_, plants without inoculum and under low-irrigation conditions died. Despite this, the root system of the plants was collected, dried, weighed and stained with trypan blue. AMF root colonization was measured because the roots maintained their consistence few days after plants died. These plants developed a total of five leaves, which is a lower value in comparison to the rest of the treatments (in which plants developed six to eight leaves).

At the end of the experiment, both the root AMF colonization and number of AMF spores showed similar trends among treatments. For highly irrigated treatments, AMF activity was significantly higher in the +B+AMF treatment. However, no differences between +AMF treatment and +B+AMF were observed under low-irrigation conditions. Regarding the non-inoculated plants, AMF activity was significantly lower in both irrigation conditions. The observed peaks in the AMF activity were probably caused by (i) some contamination coming from the other treatments or (ii) the regeneration of these microorganisms by the substrate itself.

Both the AMF root colonization and number of spores presented higher values in WID2 in comparison to WID1. This confirms the greater development of mycorrhizae under stress conditions as an adaptation strategy.

The aerial and root dry biomass values also showed significant differences between treatments: +B+AMF treatment reported the highest values for ADB under both water irrigation treatments. However, these differences between inoculated plants were only statistically significant under lower doses of water. Non-mycorrhized plants exhibited a significant decrease in ADB under well irrigation conditions with respect to AMF treatments.

The root dry biomass (RDB) values showed a clear trend under low-irrigation doses, where +B+AMF treatments led to better results. However, for highly irrigated scenarios, the Control treatment without AMF showed similar behavior to +B+AMF mycorrhized plants.

Finally, the leaves nutrient concentrations did not show any significant difference among treatments. Table S5 details the complete information of these results.

## 3. Discussion

Results for AMF root colonization and number of AMF spores in Experiment 1 confirm that biochar application had a significant influence on AMF activity. Slight differences were observed, depending on the texture of the selected substrates.

According to previous publications [22,24,25], the activity of AMF increased after biochar application. However, depending on the experimental conditions and especially on soil-nutrient availability, decreases in AMF root colonization have also been reported [18,21]. In this particular experiment, it was confirmed that biochar had a positive effect on AMF development in the sandy-loam texture (S1); nevertheless, the higher activity of mycorrhized roots or number of AMF spores did not result in any marked increase in the biological crop yield, as reported in a previous publication [30]. The obtained results in B600 treatment at high doses in the clay-loam substrate, as mentioned above, showed the minimum value of root colonization and the maximum value for the number of AMF spores, which leads to the conclusion that the sporulation strategy by the mycorrhiza prevailed in this treatment over root colonization. This statement could be related to a stress in the plant. Considering the results obtained in a previous publication [30], in which the productive parameters of this experiment were analyzed, no decrease in aerial biomass production was observed for this treatment. However, a lower number of plants fructified under this treatment, meaning that plants did not complete their productive cycle before drying.

Warnock et al. [33], assuming the importance of this community of microorganisms in their studies, elucidated the possible mechanisms that could explain how biochar could alter the total abundance of mycorrhizal fungi in soils and plant roots. In addition to the presence of available phosphorus in the soil, these mechanisms are affected by several factors: (i) modifications in the activity of linked microorganisms, (ii) changes in signaling dynamics between plants and mycorrhizal fungi, and (iii) physical effects related to biochar porosity. Regarding these mechanisms, we relate the higher influence of biochar produced at 400 °C on AMF activity to the higher volatile content of the biochar with respect to biochar produced at 600 °C. Volatile compounds could alter signaling dynamics in the soil, combined with an increase in water-holding capacity of the affected substrate (sandy-loam origin) as it was reported in a previous publication [30]. The multiplication process developed in Experiment 2, where the substrate was composed of sandy-loam soil, as well as the results obtained from Experiment 3 sustain the preliminary results of Experiment 1, where increases in AMF activity in the presence of biochar were observed. It is important to emphasize the role played by the introduction of mycotrophic crops in order to optimize the development of indigenous AMF [15], as was seen when roots of basil and sorghum were combined in the multiplication containers. The average values of root colonization in Experiment 2 were lower than those expected according to the number of AMF spores measured. By comparing these results with those reported in a previous study (which was conducted for different solid substrates [34]), one can conclude that the observed increases in root colonization (caused by biochar addition) are in line with the outcomes reported for other substrates tested (i.e., vermiculite, perlite and volcanic residues).

The observed interactions between biochar and *Pseudomonas* genus represent a positive finding of this study. These PGPR bacteria appear to be “mycorrhizal helper bacteria” [29] and they were previously studied in order to investigate their relationships with plant nutrition on two fronts: alleviating the abiotic plant stresses [35] and enhancing the host plant defenses [36]. Ordóñez [37] observed a positive interaction between these genera, increasing P acquisition by plants. Other authors determined the synergistic use of biochar and PGPR for enhancing crop growth under water-deficit conditions [38]. Due to the reduced effects of biochar application on microbial communities observed in this study, further and longer-term studies are required. According to Yadav et al. [6], the aging of biochar could play an important role by increasing microbial biomass activity due to an easier access to carbon sources that are not available in the fresh biochar.

Regarding the enzymatic activity, the results reported here could be complemented by conducting an in-depth study on phosphorus availability in the soil and pH variations along the experiment. Several studies [39,40] have already observed inverse relationships between inorganic P availability and phosphatase activity, although this was dependent on initial bioavailable P. Soil pH also affects the activity of enzymes due to the pH sensitivity of amino acid functional groups (i.e., at different pH, the conformational preference and spatial structure of amino acids can change). In addition, pH can also affect enzyme activity by influencing the concentration of inhibitors or activators in the soil solution and the effective concentration of the substrate [41]. Nevertheless, previous publications reported increases in the phosphatase activity after the application of biochar produced at high temperatures (≥500 °C) [42,43]. From the results reported above, a general trend for phosphatase behavior as a function of biochar application and application rates cannot be established. An apparent effect of B600 application was observed for the sandy-loam substrate in contrast to B400 addition and Control treatment. This influence of the high biochar temperature differs from other previous studies [44,45], in which phosphomonoesterase activity was increased to a greater extent when biochars produced at lower temperatures were applied.

The suitability of using this biochar as a component of growing media substrate in the multiplication process of AMF was demonstrated in Experiment 2. The search for alternatives to the use of *Sphagnum* peat as well as the facility to experimenting with biochar under controlled conditions results in a large number of pot-growth-based studies [27]. The physicochemical characteristics of this material have largely been related to some key processes in containerized plant production such as improvement of water retention [9,46,47,48] and utility as a potential carrier material for delivery of agrochemicals and inoculants [28]. In this sense, several factors influence the scalability of these processes, for which specific life-cycle analyses should be carried out in depth [49]. From the biotechnology perspective, a good microbial carrier should possess essential characteristics such as the capacity to deliver the suitable number of viable microbial cells at the right time [50], which has been reported in this study. Results from different studies show the potential of biochar to be used as an alternative inoculum carrier to peat and vermiculite. Hale et al. [51] tested biochar from different feedstocks and the influence of its characteristics on rhizobacteria survival. According to this study, Ghazi [52] reported a positive performance of biochar to support a *Rhizobia* inoculant in storage conditions. Various factors influence the scalability of these processes.

Biochar particle size deserves special attention in the present study, in which field conditions were simulated with a previous processing of biochar. Relatively large particle size was used for the pot experiments, with the exception of Experiment 3, in which solid substrate was grinded. One can expect a positive effect of reducing biochar particle size on all (or almost all) the parameters tested. Thus, biochar particle size appears to be an important factor that needs to be further investigated in order to explain the evolution of productive parameters as it has been studied in previous publications [53].

## 4. Materials and Methods

### 4.1. Biochar Production, Characterization and Processing

Vine shoots from winter pruning of vineyards (*Vitis vinifera* L.) were used as biochar precursor through slow pyrolysis at 400 °C (B400), and at 600 °C (B600) as the final pyrolysis temperatures. Vine shoots were cut using a domestic chipper into smaller pieces of 4–7 cm length. Pyrolysis experiments were carried out in a fixed-bed laboratory reactor. Information relative to the pyrolysis device, operating conditions and biomass/biochar characterization methodology are detailed in previous publications [30,54].

Both types of biochar were mechanically processed through an automatic agitation system [30] developed at laboratory scale to reproduce the movement in a commercial fertilizer spreader. Different particle sizes (wt. %) were obtained from this process: B400 (7% < 2 mm; 24% 2 mm < x < 20 mm; 43% 20 mm < x < 40 mm; 26% ≥ 40 mm); B600 (4% < 2 mm; 19% 2 mm < x < 20 mm; 55% 20 mm < x < 40 mm; 22% ≥ 40 mm).

### 4.2. Soils Characterization

Two different agricultural soils with contrasting textures (S1: Soil 1 Calcisol–sandy-loam; S2: Soil 2 Cambisol–clay-loam) were selected. Both soils were managed under agroecological techniques and were covered with spontaneous flora at the time of sampling. A 10-point sampling of the first 5–30 cm was performed to obtain a representative soil sample of the rhizosphere. Samples were air-dried in the laboratory and sieved through a 2 mm mesh. The methodology adopted for the physicochemical characterization of the soils is detailed in a previous publication [30].

A preliminary biological characterization was conducted and the following parameters were evaluated:

AMF infective potential. (a) The number of AMF infective propagules was quantified using the Most Probable Number (MPN) methodology [55,56], which was previously adopted by Sánchez de Prager et al. [57]. Serial dilutions of soils mixed with a sterilized substrate were established in tray fillers (60 cm^3^/filler); a mycotrophic species (barley) was selected for this experiment. Chemically untreated barley seeds were pregerminated and previously sterilized in tempered bleach solution (1 vol. %) for 30 min. One pregerminated seed per tray filler was cultivated for 6 weeks. After this period, plants’ roots were separated from the substrate and washed with water, cleared with 2.5% KOH for 24 h, acidified with 0.01% HCl, stained following the methodology proposed by Phillips and Hayman with acidified glycerol (50% glycerol, 49.95% H_2_O, 0.05% HCl) and 0.05% trypan blue [58], and cut in 1–2 cm pieces. Root colonization was quantified by the magnified intersect method proposed by McGonigle et al. [59]; (b) AMF spores were isolated from the soil samples following the procedure proposed by Gerdemann and Nicholson [60] and modified by Sieverding [56]. By the nature of the bioassay, the identification of the AMF was carried out morphologically at the genus level. A microscope at up to 400-fold magnification was used as described for glomeromycota classification by Oelh et al. [61] and Sanchez de Prager et al. [57].

Microbiological analyses. For that, 25 g were sampled of each soil. Samples were decimal diluted in 0.1% sterile distilled peptone water (Merck, Darmstadt, Germany) and homogenized using a laboratory blender Stomacher 400 Circulator (Seward Laboratory, London, England) for 2 min at 250 rpm. ISO standards were followed for each microbial group enumerated: Mesophilic aerobic microorganisms (4833–1:2014), *Pseudomonas* genus (13720:2011), and Mycobiota (21527:2008). Actinomycetes species were cultured on Starch Casein Agar (SCA) for 5–7 days at 30 °C according to Bawazir et al. [62].

### 4.3. Experimental Designs and Agronomic Tests Establishment

Three agronomic tests were carried out with different purposes:

Experiment 1 was carried out to optimize the biochar addition ratio and final pyrolysis temperature with AMF inoculum multiplication purposes. A pot-based experiment growing sorghum crop (*Sorghum bicolor* L. Moench) was conducted under controlled greenhouse conditions. Polyethylene trays with 12 conical fillers of 650 cm^3^ volume capacity, 18 cm deep and 64 cm^2^ of the upper surface were used for carrying out the bioassay. Three pregerminated seeds per tray filler were placed and carefully watered for 2 months until a thinning was performed (maintaining one plant per tray filler). Sorghum is a classic mycotrophic species widely used as a trap plant in studies and multiplication processes of AMF [63,64]. A randomized factorial block design was adopted with three factors as independent variables, as is detailed in Table 6. The duration of the trial was 13 months in which the crop completed two production cycles as is detailed in a previous publication [30].

Experiment 2. Considering the results obtained in Experiment 1, B400 was evaluated as a component of solid substrate for the multiplication process of AMF consortium of S1 (sandy-loam texture soil). The process was based on the growth of trap plants in solid substrate. A one-factor design was adopted with 2 levels for the biochar application rate. The distribution of treatments was randomized complete blocks, with 3 replicates for each one (see Table 6). The experimental unit was placed in an 8 L plastic container (40 × 20 × 16 cm) and was composed of 8 seedlings of sorghum (*Sorghum bicolor* L. Moench) as host/trap plants combined with 8 seedlings of basil (*Ocinum basilicum*), which was cut cyclically to avoid overdevelopment (all the seeds were previously sterilized and pregerminated as is described in Section 4.2.). These species were selected considering their high degree of mycorrhization and their adaptation to relatively high temperatures, since this experiment was carried out between the months of March and July (see Table S6 for more information about experimental conditions). At the end of the development cycle, the aerial part of the plants was cut and the solid substrate was processed, cutting the roots into small pieces (1–2 cm) and forming a homogeneous mixture of all components (roots, soil, fine gravel and peat, as is described in Table S6). Colonized roots and spores present in the substrate are the mycorrhizal propagules that constitute the new inoculum.

Experiment 3. The efficacy of the inoculants obtained in Experiment 2 was evaluated on a lettuce (*Lactuca sativa* var. Capitata) crop subjected to water-stress conditions. A randomized factorial block design was adopted with two factors as independent variables (see Table 6). The amount of each inoculum added was the equivalent to 50 AMF spores. One pregerminated lettuce seed was established per pot. Plastic pots with 704 cm^3^ of volume capacity (8 × 8 × 11 cm) were selected for this agronomic test.

The duration of the test was 2 months. During the first 30 days, all the treatments received similar water irrigation inputs (WID1, see Table 6) through gravimetric measurements. From the second month, water irrigation for half of the plants was progressively stopped until 10% field capacity level for the rest of the experiment (62 days).

Detailed information on the sowing process, substrates composition, growing conditions and monitoring of environmental conditions at the greenhouse is available in Table S6 of supplementary material.

### 4.4. Substrate Analysis and Plant Measurements

For Experiment 1, a detailed report of productive parameters and physicochemical changes in the substrates is collected in a previous publication [30]. Regarding the biological parameters, the following measurements were carried out during the experiment and after the first harvest:

AMF root colonization: three measurements of AMF root colonization were done at different dates after sowing (120 days–D_120_, 210 days–D_210_, and 330 days–D_330_) while the crop was established. A 3-point sampling was performed for each pot with a bipartite gouge auger (Eijkelkamp, Netherlands) 200 mm length and Ø13 mm. Plant roots were separated from substrates and washed with water before staining following the method described in Section 4.2. Direct estimation of AMF root colonization was carried out through microscopic examination following the “magnified intersection method” [58]. The final root system of each plant was processed through the same methodology after washing and drying at 70 °C to measure total root dry weight [30]; thus, final AMF root colonization was measured 390 days after sowing (D_390_). The quantification of the number of AMF spores was carried out for each pot substrate at the end of the experiment (D_390_) following the methodology detailed in Section 4.2. It is important to emphasize that no external mycorrhizal inoculum was added to the tested pots; only the indigenous mycorrhizal consortium of each soil was worked on.

Microbiological analyses: they were performed for each pot substrate at the end of the experiment (D_390_) following the methodology detailed in Section 4.2.

Enzymatic activity: AcdP and AlkP were analyzed based on the colorimetric determination of the *p*-nitrophenol (PNP) released by the enzyme. The methodology followed was proposed by Tabatabai and Bremner [65], and it was adapted at pH 5.5 for AcdP and pH 11.0 for AlkP.

For Experiment 2: root colonization at the end of the experiment was measured in a representative number of both basil and sorghum roots. Once the aerial part of the plants was cut, and before grinding the solid substrate, a portion of the central part of the root system of all the plants in each experimental unit was sampled in order to obtain root-colonization information through the stain methodology described in Section 4.2. Three samples from the solid substrate of each experimental unit were reserved for wet sieving analyses and MPN methodology described in Section 4.2.

For Experiment 3: For AMF measurements, the methodologies described in Section 4.2 were conducted at the end of the experiment. Yield was measured by cutting the plants at the end of the experiment and drying in an oven until weight stabilization at 70 °C. Shoot and roots dry weight were differentiated. The analysis of elemental N (thermic conductivity), P (spectrometry) and K (spectrometry) in leaves was conducted once yields were measured. Leaf greenness was measured three times after sowing (D_30_, D_50_, D_62_) in Experiment 3 as soil-plant analyses development (SPAD) readings (Chlorophyll Meter SPAD-502, Konica Minolta, Osaka, Japan).

### 4.5. Statistical Analysis of Results

Final data were statistically analyzed using the IBM SPSS Statistics v.26 software package. The T-Student test was conducted to analyze results from initial biological characterization and results of Experiment 2. Three-way ANOVA was conducted when the effects of factors and their interactions were studied. Two-way and one-way ANOVA were also conducted in selected cases (e.g., for different textures of growing substrate type and bifactorial experimental designs). Means comparisons were combined with Tukey´s and Scheffe´s test with a significance level of 0.05. In order to meet the criteria of statistical normality and homoscedasticity, data for the number of AMF spores and the number of infective propagules were transformed into natural logarithms, and the data of root colonization (%) were transformed into arcsine for statistical analysis.

## 5. Conclusions

The application of vine-shoots-derived biochar modifies the soil biological properties (i.e., AMF activity, microbial communities and enzymatic activity) to different extents depending on the factor being considered (texture substrate, biochar final pyrolysis temperature and application rate). The bioindicators selected in this study were affected by biochar application, despite the large particle size of the biochar used in this experiment. These indicators were more severely affected by the sandy-loam substrate compared to the clay-loam one. The suitability of using this biochar as a component of growing media substrate in the multiplication process of AMF was demonstrated. The combination of biochar and AMF could alleviate plant stress under drought conditions.

Considering the findings reported here, further specific field-scale experiments are required to understand better and assess the effects of vine-shoot-derived biochar application in soils. Results from these studies will also be useful to relate changes in the biological soil activity to physical properties (water-holding capacity, stability of soil aggregates, etc.) and productive parameters, which were slightly affected in this study.

## Figures and Tables

**Table 1:** AMF infective potential and microbiological composition of soils.

Determination ^1^	Unit	S1 ^2^	S2
AMF potential (MPN)	Number of infective mycorrhizal propagules 100 ${\mathrm{cm}}^{3}{}^{-1}$	39.6 ± 9.3	30.9 ± 13.1
Number of AMF spores	Number of AMF spores 100 g soil^−1^	420 ± 18	465 ± 23
Identified genera	% spores	20 *Gigaspora* spp. 3 *Scutellospora* spp. 77 *Glomus* spp.	6 *Acaulospora* spp. 94 *Glomus* spp.
Culturable microbial communities			
Mesophilic aerobic microorganisms	log cfu g^−1^	6.59 ± 0.90	7.08 ± 0.67
Actinomycetes	log cfu g^−1^	6.08 ± 0.45	6.45 ± 0.27
*Pseudomonas* genus	log cfu g^−1^	5.74 ± 0.13	5.96 ± 0.34
Mycobiota	log cfu g^−1^	4.75 ± 0.65	4.71 ± 0.43

^1^ Results from five replicates of each sample of soil. Average values and standard deviation. ^2^ S1—soil 1: sandy-loam; S2—soil 2: clay-loam.

**Table 2:** Effect of biochar addition on root AMF colonization and number of AMF spores (final pyrolysis temperature of biochar B400—400 °C and B600—600 °C; application rate D0—Control without biochar, D1—1.5 wt. %, D2—3 wt. %). Average values and standard deviation (in brackets).

Variable Measured		Sandy-Loam Substrate					Clay-Loam Substrate				
		D0	B400		B600		D0	B400		B600	
			D1	D2	D1	D2		D1	D2	D1	D2
Root AMF colonization %	D_120_	10.0 a (1.0)	13.3 a (5,7)	12.5 a (1.0)	10.0 a (1.5)	10.0 a (1.5)	10.5 a (2.0)	13.3 a (6.7)	10.0 a (0.0)	11.6 a (4.0)	13.3 a (5.7)
	D_210_	11.7 a (2.8)	18.3 a (5.7)	16.7 a (2.9)	11.7 a (2.8)	13.3 a (2.9)	21.7 a (5.7)	21.7 a (5.7)	13.3 a (28.9)	13.3 a (2.8)	15.0 a (0.0)
	D_330_	8.3 b (2.9)	13.3 ab (2.9)	20.0 a (0.0)	10.0 b (5.0)	11.7 ab (2.9)	26.7 ab (2.9)	26.7 ab (7.3)	31.7 a (2.9)	26.7 ab (2.9)	18.3 b (2.9)
	D_390_	5.7 bc (1.1)	13.3 ab (2.9)	18.3 a (2.9)	5.0 c (0.0)	10.0 bc (0.0)	25.0 a (0.0)	23.3 a (2.9)	31.6 a (2.9)	25.0 a (0.0)	6.7 b (5.8)
Number of AMF spores 100 g soil^−1^	D_390_	548 c (103)	830 bc (198)	1511 a (170)	1380 ab (275)	1095 ab (230)	1668 b (200)	2058 b (232)	1930 b (287)	1978 b (95)	5245 a (1399)

Means within a row followed by the same letter are not significantly different for the same kind of growing substrate at *p* ≤ 0.05 (Tukey’s test).

**Table 3:** Three-way ANOVA results on the effects of growing substrate texture, biochar temperature and application rate on root AMF colonization, number of AMF spores, phosphatase activities and microbial communities.

Factor	Root AMF Colonization			AMF Spores	Phosphatase Activity		Microbial Communities	
	D_210_	D_330_	D_390_		AcdP	AlkP	MAM	PS
Growing substrate texture (S)		<0.0001	<0.0001	<0.0001	<0.0001	<0.0001		0.040
Biochar temperature (B)		0.001	<0.0001		0.001	<0.0001		<0.0001
Application rate (D)					<0.0001	<0.0001		
S × B						0.006		0.012
S × D			0.002		0.006	<0.0001		
B × D	0.048	0.007	<0.0001	0.005	0.001			0.001
S × B × D			<0.0001	0.003	0.001		0.011	

D_210/330/390_: days after sowing; AcdP: Acid phosphatase activity: AlkP: Alkaline phosphatase activity; MAM: mesophilic aerobic microorganisms; PS: *Pseudomonas* genus.

**Table 4:** Effects of biochar addition on AMF measurements at the end of the multiplication process of an indigenous AMF consortium (T0: 0 wt. % biochar; T1: 1.5 wt. % biochar).

Treatment	AMF Root Colonization (%)	Number of AMF Spores 100 g Soil^−1^	Infective Mycorrhizal Propagules 100 ${\mathrm{cm}}^{3}{}^{-1}$
T0	15.5 ± 2.1	514.6 ± 59.3	72.4 ± 32.0
T1	32.8 *** ± 5.2	866.9 *** ± 125.0	161.5 ** ± 80.3

Data are average of six replicates for AMF root colonization (basil + sorghum) and three replicates for the rest of the parameters ± SD. Statistically significant differences at ** *p* ≤ 0.01; *** *p* ≤ 0.001.

**Table 5:** Effects of AMF inoculum addition on AMF measurements, dry biomass and the SPAD index in lettuce crop (WID1: 70–80% container capacity, WID2: 10% container capacity; −AMF: without inoculum, +AMF: with AMF inoculum, +B +AMF: with AMF inoculum obtained from a solid substrate with biochar; ADB: aerial dry biomass at the end of the experiment, RDB: root dry biomass at the end of the experiment; D_30,50,62_: days after sowing; *n*: number of living plants at the end of the experiment).

Factors		Root AMF Coloniz. (%) D_62_	Number of AMF Spores	Dry Biomass		SPAD Index			*n*
Irrigation	Inoculum			ADB	RDB	D_30_	D_50_	D_62_	
WID 1	−AMF	1.0 ± 3.0 c	2.0 ± 4.3 c	2.70 ± 0.35 ab	1.28 ± 0.20 b	27.4 ± 3.2	36.2 ± 2.1 a	37.2 ± 2.5 a	10
	+AMF	13.0 ± 3.5 b	51.8 ± 14.3 b	3.45 ± 0.50 ab	0.93 ± 0.23 b	27.5 ± 1.2	36.9 ± 2.0 a	37.24 ± 1.8 a	9
	+B +AMF	24.5 ± 7.2 a	144.1 ±35.6 ab	3.71 ± 0.48 a	1.26 ± 0.21 b	29.0 ± 1.0	35.7 ± 2.3 a	35.9 ± 2.6 a	9
WID 2	−AMF	2.0 ± 4.2 b	1.9 ± 4.2 c		1.25 ± 0.45 b	28.5 ± 3.0	21.6 ± 4.2 c		0
	+AMF	19.0 ± 10.0 a	178.6 ± 17.9 a	2.49 ± 0.21 c	1.39 ± 0.39 b	27.6 ± 3.5	28.1 ± 3.8 b	16.5 ± 1.1 c	5
	+B +AMF	24.0 ± 6.0 a	201.3 ± 73.0 a	2.69 ± 0.25 bc	1.92 ± 0.60 a	27.1 ± 4.1	33.7 ± 1.4 a	17.2 ± 1.4 b	8

Data are average of 10 replicates ± SE with except for ADB and SPAD D_62_, in which data is average of *n* ± SD. Different letters within a column denote statistically significant differences at *p* ≤ 0.05 (Scheffe’s test).

**Table 6:** Experimental designs adopted to evaluate the effects of biochar addition as a component of the solid substrate in the multiplication process of AMF.

Experiment	Factors	Treatments	Replicates
1	1. Growing media texture	S1—substrate 1—sandy-loam growing media S2—substrate—2—clay-loam growing media	Five replicates/treatment: 50 experimental units
	2. Final pyrolysis temperature	B1—biochar 1—400 °C B2—biochar 2—600 °C	
	3. Biochar application rate	D0—Control—without biochar	
		D1—1.5 wt. %	
		D2—3 wt. %	
2	1. Biochar application rate	T0—Control—without biochar	Three replicates/treatment: 6 experimental units
		T1—1.5 wt. % B1	
3	1. Inoculum composition	−AMF—Control—without inoculum	Ten replicates/treatment: 60 experimental units
		+AMF—+ inoculum obtained from T0	
		+B+AMF—+ inoculum obtained from T1	
	2. Water irrigation dose	WID1—70–80% container capacity	
		WID2—10% container capacity	

## Data Availability

The data presented in this article are available on request from the corresponding author.

## Supplementary Materials

The following are available online at https://www.mdpi.com/article/10.3390/plants10050950/s1, Table S1: Proximate, elemental and physicochemical analyses of biochar produced at two different temperatures, Table S2: Results from physicochemical fertility analyses of soils collected for the agronomic test, Table S3: Average values and standard deviation (in brackets) of the effect of growing substrate texture, biochar temperature and application rate on the identified genera of AMF, culturing microbial communities and phosphatase activity in a pot sorghum crop experiment, Table S4: Pearson correlation and p-valor (in brackets) between AMF parameters and microbial communities in a sandy-loam substrate, Table S5: Effect of AMF inoculum addition on nutrient leaves of lettuce, Table S6: Additional information to Section 4.3. Experimental designs and agronomic tests establishment.
