# Supplementary material for: The Effects of Biochar on Indigenous Arbuscular Mycorrhizae Fungi from Agroenvironments
Source: Plants (Basel). 2021 May 10;10(5):950. doi: 10.3390/plants10050950 (PMC8150396; doi:10.3390/plants10050950)
Supplement: Supplementary file 1 [file plants-10-00950-s001.zip › plants-1147178-supplementary.pdf]

The Effects of Biochar on Indigenous Arbuscular Mycorrhizae Fungi from Agroenvironments

María Videgain-Marco, Pedro Marco-Montori, Clara Martí-Dalmau, M. Carmen Jaizme-Vega, Joan Josep Manyà-Cervelló and Francisco Javier García-Ramos

SUPPLEMENTARY MATERIAL

Table S1. Proximate, elemental and physicochemical analyses of biochar produced at two different temperatures (400°C — B400 and 600 °C — B600).

**Proximate**			**Elemental (wt. % in daf[1] basis)[2]**		
	**B400**	**B600**		**B400**	**B600**
Ash (wt. % in dry basis)	6.45 ± 0.14	10.02 ± 0.81	C	71.50 ± 0.48	82.89 ± 0.33
			H	4.46 ± 0.19	1.95 ± 0.08
Moisture (wt. %)	0.57 ± 0.22	3.05 ± 0.34	N	1.58 ± 0.10	1.52 ± 0.01
			O	22.42 ± 0.77	13.63 ± 0.31
Volatile matter (wt. % in dry basis)	22.03 ± 0.01	2.39 ± 0.71	**Surface area and pore volume**		
			S_{bet} (m^2 g^{-1})	105.8	227.5
Fixed carbon (wt. % in dry basis)	70.88 ± 6.45	84.54 ± 1.32	V_{total} (cm^3 g^{-1})	0.0370	0.0819
			V_{ultra} (cm^3 g^{-1})	0.0361	0.0816

Physicochemical analyses (organic amendment reference)

**Principal nutrients**	**Method**	**Unit**	**Result**		**Secondary nutrients**	**Method**	**Unit**	**Result**	
			**B400**	**B600**				**B400**	**B600**
N_{total} Kjeldahl	MT-FER-001	wt %	1.40	1.20	Ca_{total} (CaO)	ICP-OES	wt %	2.90	3.80
P_{total} (P_2O_5)	VISIB. ULTR.	wt %	2.08	2.45	Mg_{total} (MgO)	ICP-OES	wt %	0.68	0.79
K_{total} (K_2O)	ICP-OES	wt %	1.70	2.10	Na_{total} (Na_2O)	ICP-OES	mg kg^{-1}	850.00	540.00
**Microel.**	**Method**	**Unit**	**Result B400**	**Result B600**	**Phys-Chem**	**Method**	**Unit**	**Result B400**	**Result B600**
Fe_{total}	ICP-OES	mg kg^{-1}	280	210	SOM	Calcin.	wt %	86.40	85.40
Co_{total}	ICP-OES	mg kg^{-1}	43	38	Apparent density		g $(cm^3)^{-1}$	0.33	0.40
Mn_{total}	ICP-OES	mg kg^{-1}	100	102	Soil water retention	Gravim.	v %	14.16	18.35
Zn_{total}	ICP-OES	mg kg^{-1}	145	135	pH (1:2.5)	Potenc.		8.6	8.57

[1] Dry-ash-free.

[2] Oxygen is calculated by difference.

Table S2. Results from physicochemical fertility analyses of soils collected for the agronomic test (S1—soil 1: sandy-loam; S2—soil 2: clay-loam).

Determination	Method	Unit	S1	S2
pH (1 : 2.5 water)	Potenciometry		8.0 ± 0.5	8.4 ± 0.5
Electrical conductivity (1 : 5)	Electrometry	dS m^{-1}	0.2 ± 0.03	0.2 ± 0.03
Oxidable organic matter	Espectrofotometry	wt. %	3.04 ± 0.38	1.70 ± 0.21
N (N-NO_3)	Espectrofotometry	mg kg^{-1}	59 ± 8	12 ± 2
P (Olsen)	Espectrofotometry	mg kg^{-1}	32 ± 3	29 ±3
K	AAS	mg kg^{-1}	232 ± 39	88 ± 15
Mg	AAS	mg kg^{-1}	160 ± 33	252 ± 52
Water holding capacity	Gravimetry	v %	5.41	10.22

Table S3. Average values and standard deviation (in brackets) of the effect of growing substrate texture, biochar temperature and application rate on the identified genera of AMF, culturing microbial communities and phosphatase activity in a pot sorghum crop experiment
(B400 – Biochar 400 °C; B600 – biochar 600 °C; app. rate D1 – 1.5 wt. %; app. rate D2 – 3 wt. %; G: *Gigaspora* spp.; S: *Scutellospora* spp.; G: *Glomus* spp.; A: *Acaullospora* spp.).

**Variable measured**		**Sandy-loam substrate**					**Clay-loam substrate**				
		**Control**	**B400**		**B600**		**Control**	**B400**		**B600**	
			**D1**	**D2**	**D1**	**D2**		**D1**	**D2**	**D1**	**D2**
Identified genera of AMF %		16 *G*<br>(5)<br>2 *S*<br>(2)<br>82 *G*<br>(3)	15 *G*<br>(9)<br>6 *S*<br>(4)<br>79 *G*<br>(6)	15 *G*<br>(7)<br>5 *S*<br>(3)<br>80 *G*<br>(12)	18 *G*<br>(6)<br>6 *S*<br>(4)<br>76 *G*<br>(14)	16 *G*<br>(5)<br>3 *S*<br>(0)<br>81 *G*<br>(11)	7 *A*<br>(2)<br>93 *G*<br>(3)	10 *A*<br>(3)<br>90 *G*<br>(9)	5 *A*<br>(3)<br>95 *G*<br>(8)	6 *A*<br>(1)<br>94 *G*<br>(6)	4 *A*<br>(1)<br>96 *G*<br>(4)
Microbial communities log cfu g^{-1}	MAM	7.49b<br>(0.22)	8.12a<br>(0.45)	8.48a<br>(0.31)	8.27a<br>(0.35)	7.90a<br>(0.06)	8.04<br>(0.13)	8.26<br>(0.25)	7.93<br>(0.08)	7.87<br>(0.27)	7.98<br>(0.08)
	PS	5.42b<br>(0.65)	6.49a<br>(0.14)	6.65a<br>(0.41)	6.37ab<br>(0.19)	6.38ab<br>(0.14)	6.17<br>(0.19)	5.95<br>(0.04)	5.61<br>(0.44)	5.88<br>(0.33)	5.79<br>(0.17)
	ACT	6.44<br>(0.08)	6.29<br>(0.36)	6.47<br>(0.31)	6.24<br>(0.13)	6.31<br>(0.25)	6.60<br>(0.06)	6.40<br>(0.18)	6.13<br>(0.35)	6.18<br>(0.09)	5.83<br>(0.66)
	M	4.94<br>(0.14)	5.06<br>(0.04)	5.10<br>(0.10)	4.83<br>(0.24)	4.88<br>(0.18)	4.92<br>(0.09)	4.94<br>(0.17)	5.09<br>(0.41)	4.53<br>(0.32)	4.89<br>(0.29)
	Y	3.45<br>(0.55)	3.25<br>(0.43)	3.74<br>(0.68)	3.90<br>(0.37)	3.59<br>(0.55)	3.95<br>(0.27)	4.26<br>(0.36)	3.94<br>(0.24)	3.74<br>(0.13)	3.70<br>(0.64)
Phosphatasa activity µmol h^{-1} g^{-1} dry wt.	AcP	1.22ab<br>(0.05)	1.08b<br>(0.05)	1.21ab<br>(0.06)	1.25ab<br>(0.06)	1.37a<br>(0.13)	1.54b<br>(0.15)	1.40b<br>(0.13)	1.45b<br>(0.13)	1.21b<br>(0.20)	2.21a<br>(0.29)
	AlkP	2.23c<br>(0.12)	2.45bc<br>(0.07)	3.42bc<br>(0.15)	3.12a<br>(0.11)	2.75b<br>(0.19)	3.81a<br>(0.01)	2.58d<br>(0.01)	3.67b<br>(0.01)	3.10c<br>(0.04)	3.70b<br>(0.01)

Means within a row followed by different letters are significantly different for the same kind of growing substrate at $p \leq 0.05$ (Tukey´s test).

Table S4. Pearson correlation and p-valor (in brackets) between AMF parameters and microbial communities in a sandy-loam substrate (PS: *Pseudomonas* genus; MAM: Mesophilic aerobic microorganisms).

	Number of AMF spores	Root colonization D330	Root colonization D390	PS	MAM
**Number of AMF spores**	1				
**Root colonization D_{330}**	0,444 (0.097)	1			
**Root colonization D_{390}**	0,332 (0.226)	0.775** (0.001)	1		
**PS**	0,674** (0.006)	0.545* (0.035)	0.550* (0.034)	1	
**MAM**	0,731** (0.002)	0.568* (0.027)	0.539* (0.038)	0.697** (0.004)	1

* Statistically significant at p ≤ 0.05; *** Statistically significant at p ≤ 0.01.

Table S5. Effect of AMF inoculum addition on nutrient leaves of lettuce (400°C — B400 and 600 °C — B600; S1: sandy-loam growing substrate; S2: clay-loam growing substrate).

Nutrient	WID 1			WID 2		
	**- AMF**	**+AMF**	**+B+AMF**	**- AMF**	**+AMF**	**+B+AMF**
N-leaf	1.08 ± 0.05	1.10 ± 0.01	1.10 ± 0.03	-	1.11 ± 0.04	1.10 ± 0.03
P-leaf	0.04 ± 0.01	0.04 ± 0.01	0.04 ± 0.01	-	0.05 ± 0.01	0.04 ± 0.01
K-leaf	2.20 ± 0.15	2.21 ± 0.08	2.22 ± 0.09	-	2.18 ± 0.12	2.23 ± 0.06

Table S6. Additional information to section 4.3. Experimental designs and agronomic tests establishment.

Experiment	Additional information
1	- Hewitt nutritive solution with minimum phosphorus concentration (P^{-}) was added to water the plants (composition 1 L: 0.4044 g NO3K; 0.9446 g NO3Ca·4H2O; 0.3697 g SO4Mg·7H2O; 0.027 g PO4H2K; 0.0421 g Na EDTA-Fe; 0.00223 g SO4Mn·4H2O; 0.00309 g BO3H3; 0.000288 g SO4Zn·2H2O, and 0.00025 g SO4Cu·5H2O). The last 15 days of the bioassay, the plants were not irrigated, with the aim of stressing the crop and favoring the production of AMF spores.<br>- Sorghum crop completed two production cycles. Since sorghum is a crop with re-sprouting capacity, plants were cut about 2 cm from the growing substrate surface at 92 days after sowing. At that time, basil plants were allowed to grow until new sorghum leaves exceeded the height of basil. 156 days after sowing, plants were cut and substrates separated from the containers. Each substrate was processed cutting sorghum and basil roots in 1-2 cm pieces and remixing with the solid substrate itself; large biochar fragments (> 2 cm) still presents in the mixture were grinded up to sizes smaller than 5 mm. Solid substrates samples were stored in plastic jars for analysis and use in Experiment 3.<br>- Both soil types were mixed with sterilized fine gravel (60:40 v/v) to avoid soil compaction in the containers forming two final mixtures in which biochar was homogeneously incorporated. The proportions of particle size obtained after the mechanical processing explained in section 4.1 were maintained.<br>- Temperature and relative humidity were monitored with a sensor HOBO Pro v2 (Onset, Boune, MA, USA) installed at the plants level. It allowed to adjust the intervals and irrigation rates during the bioassay, considering crop evapotranspiration. Temperatures in this experiment ranged from 2.6 °C minimum winter temperatures to 37.0 °C maximum summer temperatures. Relative humidity ranged from 33.4% to 90.0%. Greenhouse was naturally lighted, with regular variations throughout the year from 9h light in winter months to 15 h light in summer months.
2	- The composition of solid substrates in treatments T0 and T1 was as follows: T1 (40% w/w soil S1; 40% w/w fine gravel; 20% w/w peat substrate: 25% white peat/ 55% black peat/ 17% coconut fiber/ 3% pearlite); T2 (40% w/w soil S1; 38.5% w/w fine gravel; 1.5% w/w B400; 20% peat substrate).<br>- Fine gravel was washed with water and sterilized in autoclave for 20 min. In order to eliminate possible mycorrhizal propagules in peat substrate, it was autoclaved under flowing steam conditions during 1 h and three consecutive days.<br>- Hewitt nutritive solution was added as described for Experiment 1 for all the irrigation events.<br>- Temperature and relative humidity were monitored as described for Experiment 1.<br>- Temperatures in this experiment ranged from 4.5 °C minimum spring temperatures to 42.0 °C maximum summer temperatures. Relative humidity ranged from 41.3% to 92.0%. Greenhouse was naturally lighted, with regular variations throughout the year from 11h light in March to 15 h light in July.
3	- Growing media composition was as follows: -AMF: 50% sterile soil S1 + 50% sterile fine gravel (to avoid compaction) + 1 ml filtered S1 solution to restitute microbial activity; +AMF: 50% soil S1 + 50% sterile fine gravel + AMF inoculum obtained from T0 in Experiment 2; +B+AMF: 50% soil S1 + 50% sterile fine gravel + AMF inoculum obtained from T1 in Experiment 2.<br>- Hewitt nutritive solution was added as described for Experiment 1 but, in this experiment, it was added only in the irrigation events in which all the treatments were irrigated, and the same amount of nutrients was maintained for all the treatments.<br>- Temperature and relative humidity were monitored as described for Experiment 1.<br>- Temperatures in this experiment ranged from 16.0 °C minimum summer temperatures to 41.3 °C maximum summer temperatures. Relative humidity ranged from 59.7% to 92.0%. Greenhouse was naturally lighted, with regular variations throughout the year from 14h light at the beginning of the experiment to 13 h light at the end of the experiment.
